# Fecal metabolomics combined with 16S rRNA gene sequencing to analyze the effect of Jiaotai pill intervention in type 2 diabetes mellitus rats

**DOI:** 10.3389/fnut.2023.1135343

**Published:** 2023-05-09

**Authors:** Jing Liu, Xu Wang, Qiyao Li, Chengyu Piao, Zuowang Fan, Yao Zhang, Saisai Yang, Xiuhong Wu

**Affiliations:** ^1^School of Pharmacy, Heilongjiang University of Chinese Medicine, Harbin, China; ^2^Good Laboratory Practice of Drug, Heilongjiang University of Chinese Medicine, Harbin, China

**Keywords:** Jiaotai pill, type 2 diabetes mellitus, 16S rRNA gene sequencing, UPLC-Q-exactive focus MS, metabolomics

## Abstract

The occurrence and development of type 2 diabetes mellitus (T2DM) are closely related to gut microbiota. Jiaotai pill (JTP) is used to treat type 2 diabetes mellitus, with definite efficacy in clinical practice. However, it is not clear whether the therapeutic effect is produced by regulating the changes in gut microbiota and its metabolism. In this study, T2DM rat models were established by a high-fat diet and low-dose streptozotocin (STZ). Based on the pharmacodynamic evaluation, the mechanism of JTP in the treatment of type 2 diabetes mellitus was investigated by fecal metabolism and 16S rRNA gene sequencing. The results showed that JTP decreased blood glucose (FBG, HbA1c) and blood lipid (TC, TG, and LDL) levels and alleviated insulin resistance (FINS, IL-10) in T2DM rats. 16S rRNA gene sequencing results revealed that JTP increased microbiota diversity and reversed the disorder of gut microbiota in T2DM rats, and therefore achieved the therapeutic effect in T2DM. JTP regulated 13 differential flora, which were Actinobacteria, Bacteroidetes, Firmicutes, Proteobacteria, Eubacteriaceae, Prevotellaceae, Ruminococcaceae, Clostridium_IV, Clostridium_XlVa, *Eubacterium*, *Fusicatenibacter*, *Romboutsia*, and *Roseburia*. Metabolomics analysis showed that JTP interfered with 13 biomarkers to play a therapeutic role in type 2 diabetes mellitus. They were L-Valine, Choline, L-Aspartic acid, Serotonin, L-Lysine, L-Histidine, 3-Hydroxybutyric acid, Pyruvic acid, N-Acetylornithine, Arachidonic acid, L-Tryptophan, L-Alanine, and L-Methionine. KEGG metabolic pathway analysis of the above differential metabolites and gut microbiota by using the MetaboAnalyst database and Picrust software. It was found that JTP treated type 2 diabetes mellitus by affecting metabolic pathways such as amino acid metabolism, carbohydrate metabolism, and lipid metabolism. Spearman correlation analysis revealed high correlations for 7 pharmacological indicators, 12 biomarkers, and 11 gut microbiota. In this study, the therapeutic effect and potential mechanism of JTP on type 2 diabetes mellitus were preliminarily demonstrated by gut microbiota and metabolomics, which could provide a theoretical basis for the treatment of T2DM with JTP.

## 1. Introduction

Type 2 diabetes mellitus is the most common metabolic disease (1). With the development of the social economy, the change in people’s lifestyles (increased energy intake and reduced exercise, etc.), and the aging of the population, the incidence morbidity of type 2 diabetes mellitus is increasing year by year worldwide and has become one of the biggest killers of human health (2). Glucose and lipid metabolism disorders are the main features of type 2 diabetes mellitus, and glucose and lipid metabolism disorders are closely related to the composition of the gut microbiota (3). Studies have confirmed that glucose and lipid metabolism disorders can lead to gut microbiota imbalance, while gut microbiota imbalance aggravates glucose and lipid metabolism disorders (4). The structure of gut microbiota in type 2 diabetes mellitus is significantly different compared to healthy subjects. Gut microbiota imbalance and activation of toll-like receptor 4/stress-activated protein kinase leads to insulin resistance (5). These observations highlight that gut microbiota may be a novel diagnostic and therapeutic target for the treatment of type 2 diabetes mellitus.

Traditional Chinese medicine (TCM) has accumulated a wealth of experience in the treatment of diabetes in thousands of years of clinical practice, and TCM can reduce blood glucose through multiple pathways and multiple targets, with obvious advantages in the treatment of diabetes and its complications. Increasing evidence suggests a close association between TCM and gut microbes, which interact with each other. TCM intervenes in intestinal microecology by regulating the number and proportion of gut microbiota, inflammatory factors, signaling pathways, genes, etc., which has the effects of regulating gut microbiota, protecting the intestinal mucosal barrier, restoring intestinal microbial diversity, and enhancing immune function. Intestinal microecology also affects the metabolism and absorption of TCM in the body, which can enhance or reduce the efficacy and change the toxicity of herbal medicines. Jiaotai Pill was first recorded in “Han’s Medical Circular,” and consists of Coptis chinensis, and Cinnamon. It is clinically effective in the treatment of type 2 diabetes mellitus. Modern research has found that Coptis chinensis significantly inhibits the growth of pathogenic bacteria and promotes the growth of beneficial bacteria (6, 7). Meanwhile, gut microbiota converts berberine and safranine in Coptis chinensis into hydrogenated products and the palmatine in Coptis chinensis into demethoxylated products, making it easier to be absorbed and to exert therapeutic effects (8, 9). The cinnamic acid and cinnamic aldehyde in Cinnamon can regulate the imbalance of gut microbiota (10, 11). Therefore, it is speculated that JTP can play a role in the treatment of diabetes by regulating changes in gut microbiota.

The gut microbiota is both a player and a regulator of metabolic processes (12). The metabolism of the host is not only regulated by its genome, but also by symbiotic bacteria. Metabolomics can efficiently screen biomarkers and deeply analyze the molecular mechanisms of host health or disease. Combined metabolomics with microbiomics, a scientific language explaining the effectiveness of TCM has been established, and it has a good guiding role in the diagnosis and treatment of clinical diseases (13, 14).

In this study, based on the pharmacodynamic evaluation of the efficacy of JTP in the treatment of type 2 diabetes mellitus, we performed untargeted metabolomics and 16S rRNA gene sequencing studies on stool samples to investigate the changes in endogenous metabolites and intestinal bacteria during the treatment of type 2 diabetes mellitus with JTP. Then, the relationship between host phenotype, intestinal microbiota, and metabolites was analyzed by calculating Spearman correlation coefficients. To investigate the mechanism of JTP for the treatment of type 2 diabetes mellitus from the perspective of multi-level integration of biomarkers and gut microbiota.

## 2. Materials and methods

### 2.1. Preparation of drugs

Coptis chinensis and Cinnamon were purchased from the Harbin Branch of Beijing Tong Ren Tang Pharmacy and identified as the dried rhizome of *Coptis chinensis* Franch of Buttercup family and the dried bark of *Cinnamomi cortex* Presl of Camphoraceous family by the Teaching and Research Department of Chinese Medicine Identification of Heilongjiang University of Chinese Medicine, respectively.

According to the ratio of Coptis chinensis: Cinnamon (10:1), weigh 500 g of Coptis chinensis and 50 g of Cinnamon, decoct and extract twice, add 10 times the amount of water each time, decoct for 40 min. The two decoctions were combined, concentrated and freeze-dried. A total of 88 g of lyophilized powder was obtained, the powder yield was 16%. Metformin hydrochloride was purchased from Shanghai Yuanye Biotechnology Co., Ltd. (batch number: 201812161).

### 2.2. Animals and experimental design

In this study, 32 men SD rats, weighing 150 ± 20 g, 7–8 weeks old, provided by Liaoning Changsheng Biotechnology Co., Ltd. were used, license number: SCXK (Liao) 2020–0001. The rats were housed in an SPF class animal room with a room temperature of 25 ± 1°C, relative humidity of 50 ± 5%, and a light cycle of 12 h light/night. Rats were acclimatized for a week with a standard rodent diet, and water was available *ad libitum*. The level of fasting blood glucose (FBG) and body mass were measured. Rats with normal blood glucose values were screened and randomly divided into four groups, which were the control group (Con), model group (Mod), metformin group (MET), and Jiaotai Pill group (JTP). The model and treatment groups were fed a high-fat diet for 4 weeks with two consecutive injections of 25 mg/kg of STZ (Sigma Corporation, lot number NO. 18888664). The control group was fed a conventional maintenance diet for 4 weeks and injected with an equal volume of citrate buffer intraperitoneally. After 1 week, blood glucose was stabilized, Jiaotai Pill (6.8 g of raw drug/kg) was gavaged in the JTP group, metformin (0.2 g/kg) was gavaged once daily for 10 weeks in the metformin group, and an equal volume of distilled water was gavaged in the remaining groups. All experiments were performed following the Declaration of Helsinki and were approved by the Animal Health and Ethics Committee of Heilongjiang University of Chinese Medicine (2021012709).

### 2.3. Determination of FBG, TC, TG, LDL, HbA1c, FINS, and IL-10 in serum

After the rats fasted for 12 h but water *ad libitum*, blood was taken by the tail break method, and the level of fasting blood glucose (FBG) was measured.

Blood was taken from the abdominal aorta of the rats and centrifuged at 3,000 rpm for 10 min at 4°C to collect serum. Total cholesterol (TC), Triglyceride (TG), and Low-density lipoprotein (LDL) in rat serum were measured by an automated biochemical instrument. The enzyme-linked immunoassay was used to detect glycosylated hemoglobin (HbA1c), fasting serum insulin level (FINS), and interleukin 10 (IL-10) in rats.

### 2.4. Histopathology analysis of rat pancreas

After blood collection, the rat pancreas was carefully separated and the pancreatic tissues were immersed in 10% neutral-buffered formalin to prepare pathological sections.

### 2.5. Fecal DNA extraction and high-throughput 16S rRNA sequencing

The feces of rats were collected by stimulated defecation and placed into sterilized 2 ml EP tubes. The collected feces samples were uniformly sent to Wuhan UW Medical Laboratory Co., Ltd. for testing, and the Illumina Miseq platform (Illumina Inc., San Diego, CA, USA) was used for standard bioinformatic analysis of the bacterial 16S rRNA V3-V4 region. The main procedure is as follows: take 30 ng of qualified genomic DNA samples and corresponding fusion primers to configure the PCR reaction system, set the PCR reaction parameters for PCR amplification, use Agencourt AMPure XP magnetic beads to purify the PCR amplification products, dissolve them in Elution Buffer, label them, and complete the library construction. The libraries were tested for fragment range and concentration using an Agilent 2100 Bioanalyzer. The libraries that passed the assay were selected for sequencing on the HiSeq platform (Illumina) according to the insert size.

### 2.6. Fecal metabolomics

The collected fecal samples were weighed about 100 mg, placed in 2 ml centrifuge tubes, and thawed on ice. Added 500 μL of ultrapure water, extracted by ultrasonication at 4°C for 5 min, vortex shaking for 30 s, centrifuged at 10,000 rpm for 15 min at 4°C. A total of 300 μL of supernatant was placed in 1.5 ml centrifuge tubes as the first step of extraction. Discard the remaining supernatant in the fecal sediment, add 500 μL of methanol, ultrasonic extract at 4°C for 5 min, vortex shake for 30 s, and centrifuge at 10,000 rpm for 15 min at 4°C. A total of 300 μL of supernatant was taken as the second extraction solution, and the two extracts were combined. After vortex shaking for 30 s and filtration using a 0.22 μm filter membrane, the samples were transferred to the injection vial and all samples were analyzed in positive and negative ion modes. A total of 10 mg of each sample to be tested was mixed and then processed as QC samples according to the pre-treatment method described above.

Chromatographic separation was performed on a Thermo Scientific™ Ultra Performance Liquid Chromatograph (Thermo Fisher Scientific, Waltham, MA, USA), We used an Acquity HSS T3 (1.8 μm, 100 mm × 2.1 mm; Waters, USA) at 40°C, The mobile phase was optimized, and 0.1% formic acid in water and 0.1% formic acid in acetonitrile was selected as mobile phases A and B, respectively. The injection volume was 1 μL in both positive and negative ion modes. The mobile phases were used at a flow rate of 0.4 ml/min with a gradient of 0–3 min at 1.0–10.0% B, 3–5 min at 10.0–20.0% B, 5–8.5 min at 20.0–40.0% B, 8.5–13.5 min at 40.0–99.0% B, 13.5–14 min at 99.0% B, 14–15 min at 99.0–1.0% B.

Mass spectrometry (MS) data were acquired by using Q-Exactive focus mass spectrometer (Thermo Fisher Inc., Waltham, MA, USA) in both positive and negative ion modes. The optimized conditions were as follows: spray voltage was set to 3,500/3,200 V (±), capillary ion transport temperature was set to 350°C, sheath gas flow rate was 45 arb, the auxiliary gas flow rate was 15 arb, capillary (ion transport) temperature was set to 320°C, S-lens voltage was set to 75. The full scan range was set to 60–900 m/z, the resolution was set to 70,000 FWHM on MS^1^ and 17,500 FWHM on MS^2^, the AGC target was set to 1E^6^ on MS^1^ and 2E^5^ on MS^2^, maximum allowed ion injection time was set to 100 ms on MS^1^ and 50 ms on MS^2^, Top three ddms2 for identification, isolation window was set to 1.5 m/z, secondary mass spectrometry collision energy was set to 20, 40, and 60; vertex excitation was 4–8 s, dynamic exclusion was 8 s, acquisition data type was profile mode. The multivariate statistical analysis was used to qualify the potential biomarkers in both positive and negative ion modes.

### 2.7. Statistical analysis

#### 2.7.1. Gut microbiota analysis

The spliced Tags were clustered into Operational taxonomic unit (OTUs) using the software USEARCH (v7.0.1090_i86linux32). Sequence Variants (ASVs), ASVs are sequences that are 100% similar. In turn, the feature table (Feature, a collective term for ASVs/ASVs, etc.) is obtained. The RDP classifier Bayesian algorithm was used to analyze the OTU representative sequences taxonomically, and the cluster composition of each sample was counted. The function of the microbiota was predicted using Picrust software.

#### 2.7.2. Metabolomics analysis

Raw data were analyzed using Compound Discoverer 3.2 (CD, Waltham, MA, USA) for matching and identification of differential biomarkers. Between-group difference analysis was performed using SIMCA-P (version 14.1, Umetrics, Umea, Sweden). Metabolic Pathway Analysis was performed by using MetaboAnalyst 5.0.^1^

Spearman correlation was used to analyze the association of differential biomarkers with differential gut microbiota.

Data in a normal distribution were expressed as (mean ± SD). Student’s *t*-test or Mann-Whitney *U*-test was performed to test the differences between the two groups. Statistical analyses were performed using SPSS Statistics (version.20.0; SPSS Inc., Chicago, IL, USA), and *p* < 0.05 were considered significant.

## 3. Results

### 3.1. Rat FBG results

**FIGURE 1:**
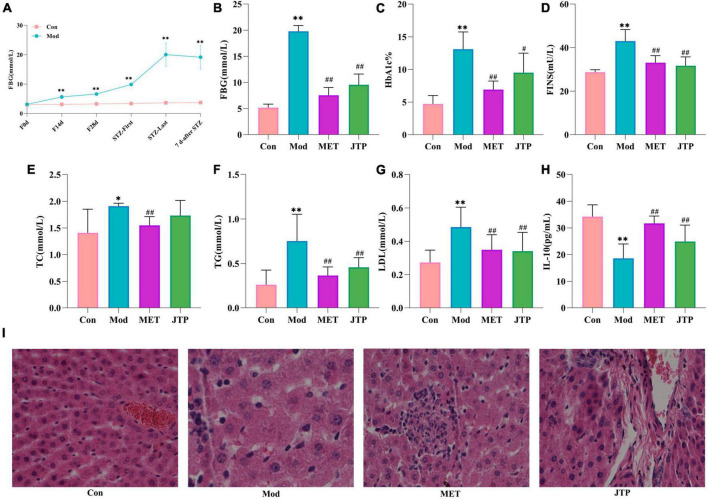
**(A)** Trends in fasting blood glucose (FBG) values in the control and model groups during the establishment of the type 2 diabetes mellitus (T2DM) model. *p < 0.05, **p < 0.01 compared to the control group (*n* = 8). **(B–H)** Changes in FBG, HbA1c, FINS, TC, TG, LDL, and IL-10 in the control, model, MET, and JTP groups (*n* = 8). *p < 0.05, **p < 0.01 compared to the control group, ^#^*p* < 0.05, ^##^*p* < 0.01 compared to the model group. **(I)** HE staining results of pancreatic tissue in the control, model, MET, and JTP groups (200×).

During the model establishment period, the fasting blood glucose (FBG) of rats showed an increasing trend in the high-fat diet stage. Compared with the control group, the FBG of the T2DM model group was significantly increased after the first injection of STZ (*p* < 0.01), and the FBG was significantly higher than 11.1 mmol/L after the second injection of STZ. After 7 days, the FBG of T2DM rats tended to be stable and reached the evaluation criteria of the type 2 diabetes mellitus model (Figure 1A).

After treatment, compared with the control group, the FBG level in the model group was significantly higher (*p* < 0.01). FBG in the metformin group (MET) and Jiaotai pill (JTP) groups was significantly lower than that in the model group (*p* < 0.01) (Figure 1B).

### 3.2. Serum levels of HbA1c, FINS, TC, TG, LDL, and IL-10

Compared with the control group, the serum levels of HbA1c, FINS, TG, and LDL were highly significantly increased (*p* < 0.01), TC levels were significantly increased (*p* < 0.05), and IL-10 levels were highly significantly decreased (*p* < 0.01) in the T2DM model rats.

Compared with the model group, the serum levels of FINS, TG, and LDL in the JTP group rats were highly significantly lower (*p* < 0.01), the level of TC had a decreasing trend, the level of HbA1c was significantly lower (*p* < 0.05), and the level of IL-10 was highly significant higher (*p* < 0.01), the levels of HbA1c, FINS, TC, TG, and LDL in the MET group were highly significantly lower (*p* < 0.01), and IL-10 levels were highly significantly increased (*p* < 0.01) (Figures 1C–H).

### 3.3. Histopathology analysis of rat pancreas

In the control group, the islets had clear boundaries, regular morphology, neatly arranged cells, and uniform size, while in the type 2 diabetes mellitus (T2DM) model group, the islets had fewer cells, disorganized cell bodies, irregular morphology, most nuclei of unequal size, and vacuolar degeneration. Compared with the model group, the islets in metformin group (MET) and Jiaotai pill (JTP) groups were observed to have a regular shape, more β-cells, neat edges, and clear boundaries (Figure 1I).

### 3.4. High-throughput sequencing of 16S rRNA

**FIGURE 2:**
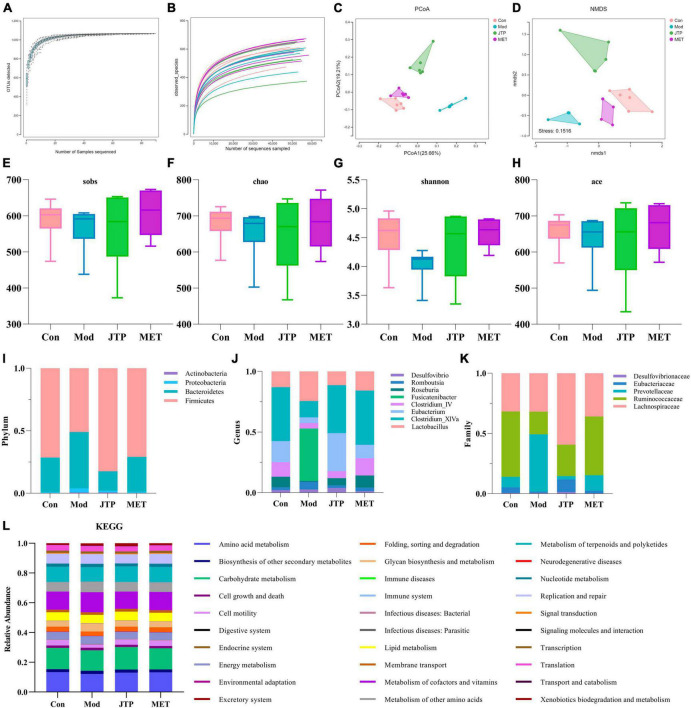
**(A)** The species accumulation curves. **(B)** Species dilution curve. **(C)** PCoA analysis of each group *n* = 6. **(D)** NMDS analysis of each group *n* = 6. **(E)** Sobs indexes *n* = 6. **(F)** Chao indexes *n* = 6. **(G)** Shannon indexes *n* = 6. **(H)** Ace indexes *n* = 6. **(I)** Differences in the abundance of bacteria in each group at the phylum level. Abscissa is sample grouping and ordinate is the relative abundance of annotated species. **(J)** Differences in the abundance of bacteria in each group at the family level. Abscissa is sample grouping and ordinate is the relative abundance of annotated species. **(K)** Differences in the abundance of bacteria in each group at the genus level. Abscissa is sample grouping and ordinate is the relative abundance of annotated species. **(L)** PICRUST analysis to predict the function of gut microbiota based on the KEGG database. PCoA, principal coordinates analysis; NMDS, non-metric multidimensional scaling.

In this experiment, based on the 97% similarity OTU obtained, the trends of the species accumulation curve (Figure 2A) and species dilution curve (Figure 2B) showed that the sequencing results had high abundance and uniform species distribution, which were sufficient to reflect the microbial information in all samples, and the sequencing data were reasonable for data analysis. As shown in the figure, sobs (Figure 2E), Chao (Figure 2F), Shannon (Figure 2G), and ace (Figure 2H) index analysis showed that the microbial community richness and microbial community diversity decreased in the model group compared with the control group, and the microbial community richness showed an increasing trend after JTP treatment. In addition, we investigated the similarity of the overall microbial community structure by β-diversity. The two axes of variation (PCo1 and PCo2) accounted for 25.66 and 19.21% of the total variance of PCoA, respectively. And the PCoA plots exhibited clear separation and obvious spatial clustering (Figure 2C). In addition, similar observations were obtained in the Non-metric multidimensional scaling (NMDS) analysis. The JTP intervention shifted the microbial community structure from the model group to the control group (Figure 2D).

At the phylum level (Figure 2I), the relative abundance of Actinobacteria, Proteobacteria, and Bacteroidetes was increased and the relative abundance of Firmicutes was decreased in the T2DM model group compared to the control group. The relative abundance of T2DM-induced disruptions in bacterial phylum levels was reversed after JTP treatment. At the genus level (Figure 2J), the relative abundances of Desulfovibrio, Romboutsia, Fusicatenibacter, and Lactobacillus were higher, and the relative abundances of Roseburia, Clostridium_IV, Eubacterium, Clostridium_XlVa were lower in the T2DM model group compared to the control group. JTP significantly reduced the relative abundance of Romboutsia and Fusicatenibacter and significantly enriched Roseburia, Clostridium_IV, Eubacterium, and Clostridium_XlVa. At the family level (Figure 2K), the relative abundance of Desulfovibrionaceae, Prevotellaceae, and Lachnospiraceae was elevated in the T2DM model group and the relative abundance of Eubacteriaceae and Ruminococcaceae was reduced compared to the control group. JTP significantly reduced the relative abundance of Prevotellaceae and had a significant enrichment effect on Ruminococcaceae and Eubacteriaceae.

We performed PICRUST analysis to predict the function of gut microbiota based on the KEGG database. The relative abundance of 30 pathways was predicted. The top ten pathways were Carbohydrate metabolism, Amino acid metabolism, Metabolism of cofactors and vitamins, Metabolism of terpenoids and polyketides, Replication and repair, Metabolism of other amino acids, Lipid metabolism, Energy metabolism, Cell motility, and Glycan biosynthesis and metabolism. And among them, the expression of Cell motility in the model group was lower than that of the control group. A callback trend was observed after the intervention with JTP. The expression of Carbohydrate metabolism, Amino acid metabolism, Metabolism of cofactors and vitamins, Metabolism of terpenoids and polyketides, Replication and repair, Metabolism of other amino acids, Lipid metabolism, Energy metabolism, Cell motility, and Glycan biosynthesis and metabolism were enriched in the model group. After JTP intervention reversed the abundance of Metabolism of cofactors and vitamins, Replication and repair, Metabolism of other amino acids, Energy metabolism, Glycan biosynthesis and metabolism (Figure 2L).

### 3.5. Fecal metabolomics

Total Ion Chromatography (TIC) of quality control (QC) samples were obtained in both positive and negative ion modes, consistently showing good peak shape and relatively uniform distribution, thus verifying that the UPLC-Q-Exactive focus was stable throughout the detection process (Supplementary Figure 1).

**FIGURE 3:**
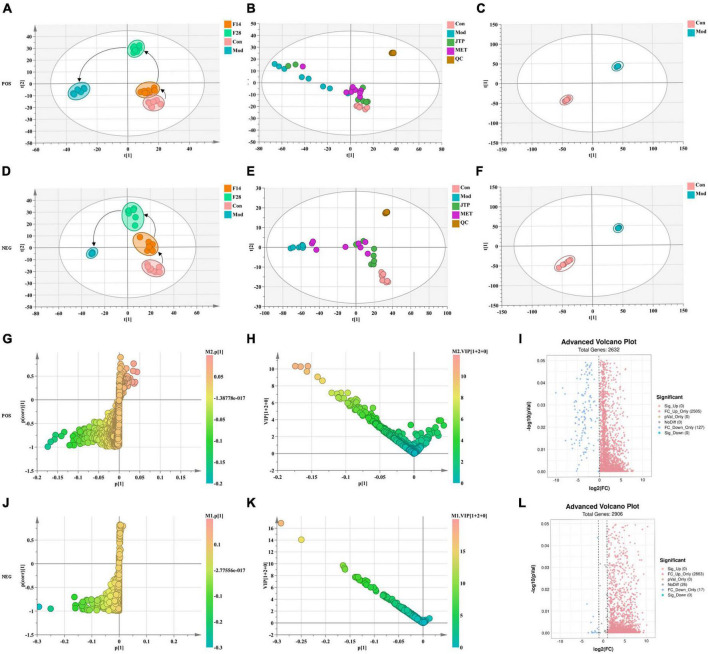
**(A)** Metabolic profile during type 2 diabetes mellitus (T2DM) modeling in positive ion mode. **(B)** Metabolic profile after treatment positive ion mode. **(C)** OPLS-DA score plots for the control group and the model group in positive ion mode. **(D)** Metabolic profile during T2DM modeling in negative ion mode. **(E)** Metabolic profile after treatment negative ion mode. **(F)** OPLS-DA score plots for the control group and the model group in negative ion mode. **(G)** Fecal biomarkers in the S-plot between the control group and the model group in positive ion mode. **(H)** Fecal biomarkers in the VIP between the control group and the model group in positive ion mode. **(I)** Fecal biomarkers in the FC between the control group and the model group in positive ion mode. **(J)** Fecal biomarkers in the S-plot between the control group and the model group in negative ion mode. **(K)** Fecal biomarkers in the VIP between the control group and the model group in negative ion mode. **(L)** Fecal biomarkers in the FC between the control group and the model group in negative ion mode.

The data of the fecal metabolic profile of rats at key time points (0, 14, 28, and 35 days) in the T2DM model replication cycle were analyzed, and a certain grouping of model and control rats appeared from day 14 of modeling, but there was an intersection and a significant difference between day 35 of modeling and day 0. This indicates that endogenous substances were changed during the transformation of healthy rats into T2DM rats (Figures 3A, D). Analyzing the metabolic profiles of each group after treatment, the JTP and MET groups were closer to the control group. QC proved that the method has good stability and repeatability, and the obtained data are reliable (Figures 3B, E).

**FIGURE 4:**
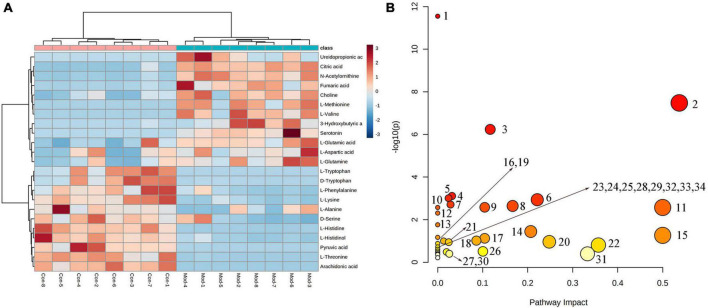
**(A)** Heatmap of changes in levels of 23 potential biomarkers in control and model groups. **(B)** Metabolism pathway analysis of 23 potential biomarkers with MetPA. MetPA, metabolic pathway analysis.

To better reveal the differences between the control and T2DM model group, Orthogonal partial least squares discrimination analysis (OPLS-DA) analysis [ESI^+^: R^2^Y-0.881, Q^2^-0.779; ESI^–^: R^2^Y-1.000, Q^2^-0.981 (Supplementary Figure 2)] was used to show that both the T2DM model group and the control group clustered significantly and there was a clear separation between the groups (Figures 3C, F). The data of control and T2DM model groups were further analyzed to obtain S-splot loading plots (Figures 3G, J), Variable important in projection plots (Figures 3H, K), and volcano plots (Figures 3I, L), and finally, the ions with VIP > 1, *p* < 0.05, and FC > 1.2 were selected as Candidate biomarkers. A total of 23 potential biomarkers in T2DM rats were finally identified (Figure 4A and Supplementary Tables 1, 2), 11 in positive ion mode, namely: L-Phenylalanine, L-Valine, Choline, L-Aspartic acid, Serotonin, L-Histidinol, Ureidopropionic acid, D-Serine, D-Tryptophan, L-Glutamine, and L-Lysine. A total of 12 in the negative ion mode, namely: L-Histidine, L-Threonine, L-Glutamic acid, 3-Hydroxybutyric acid, Pyruvic acid, Citric acid, N-Acetylornithine, Fumaric acid, Arachidonic acid, L-Tryptophan, L-Alanine, and L-Methionine. It involved 34 metabolic pathways associated with type 2 diabetes mellitus (Figure 4B and Supplementary Table 3), including 13 with impact >0.1000, which were: Alanine, aspartate and glutamate metabolism, D-Glutamine and D-glutamate metabolism, Phenylalanine, tyrosine and tryptophan biosynthesis, Phenylalanine metabolism, Arachidonic acid metabolism, Tryptophan metabolism, Histidine metabolism, Pyruvate metabolism, Citrate cycle (TCA cycle), Arginine biosynthesis, beta-Alanine metabolism, Cysteine, and methionine metabolism, Glycolysis/Gluconeogenesis.

**FIGURE 5:**
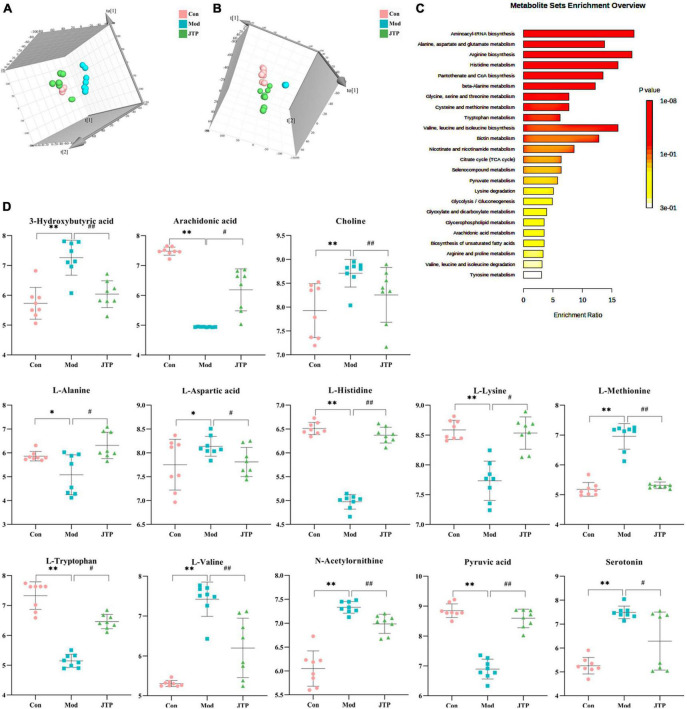
**(A)** PLS-DA analysis of the control, model, and JTP groups in positive ion mode. **(B)** PCA analysis of the control, model, and JTP groups in negative ion mode. **(C)** Metabolism pathway analysis of 13 biomarkers of JTP back regulation with MetPA. **(D)** Scatter plots of the changes in the 13 biomarkers of JTP back regulation in the control, model, and JTP groups, *p < 0.05, *p < 0.01 compared to the control group, ^#^*p* < 0.05, ^##^*p* < 0.01 compared to the model group (*n* = 8).

Partial least squares discrimination analysis (PLS-DA) analysis [ESI^+^: R^2^Y-0.995, Q^2^-0.973; ESI^–^: R^2^Y-0.952, Q^2^-0.900 (Supplementary Figure 3)] of the control, model, and JTP groups on the last day of drug administration showed that the fecal metabolic profiles of the JTP group were significantly farther from the model group and closer to the control group (Figures 5A, B). The results indicated that JTP could significantly regulate the metabolic profile of T2DM model rats to a healthy state, which further suggested that JTP could interfere with the occurrence and development of T2DM. Based on 23 potential biomarkers, the statistical analysis identified 21 of these biomarkers in the JTP callback and 13 were statistically significant (Figure 5D). JTP significantly modulates 8 of these metabolic pathways, namely: Arachidonic acid metabolism, Tryptophan metabolism, Alanine, aspartate and glutamate metabolism, Histidine metabolism, Pyruvate metabolism, Cysteine and methionine metabolism, Glycolysis/Gluconeogenesis, and Citrate cycle (TCA cycle) (Figure 5C).

### 3.6. Correlation analysis for gut microbiota, biomarkers, and pharmacological indices

**FIGURE 6:**
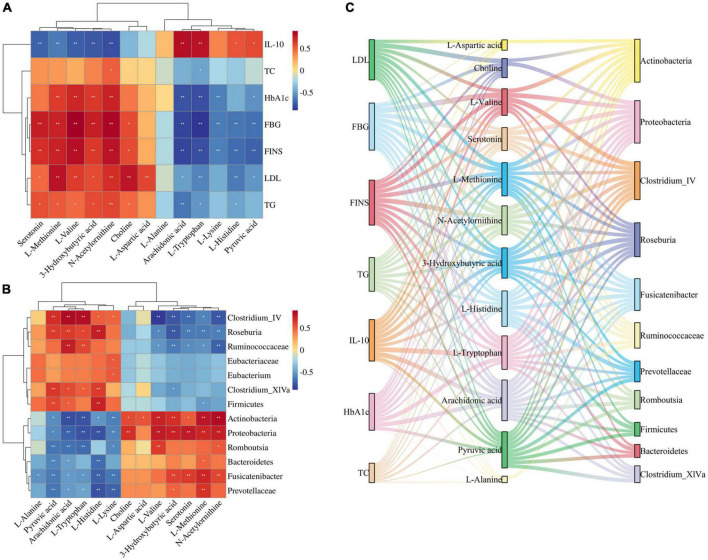
**(A)** Spearman correlation analysis of biomarkers and pharmacological indices. **(B)** Spearman correlation analysis of biomarkers and gut microbiota. **(C)** Sankey diagrams for the interconnection of pharmacological indicators, biomarkers, and gut microbiota.

Correlations were calculated using spearman for thirteen Jiaotai pill (JTP)-regulated potential biomarkers and the seven pharmacological indicators (Figure 6A). Positive correlations are shown in red, negative correlations in blue, and those with *p* < 0.05 and | r| > 0.5 were selected as correlating.

3-Hydroxybutyric acid was positively correlated with FBG, HbA1c, FINS, TG, LDL and negatively correlated with IL-10. Arachidonic acid was positively correlated with IL-10 and negatively correlated with FBG, FINS, HbA1c, and TG. Choline was positively correlated with LDL, FBG, and FINS. L-Aspartic acid was positively correlated with LDL. L-Histidine was positively correlated with IL-10 and negatively correlated with FINS, FBG, and LDL. L-Methionine was positively correlated with LDL, FINS, FBG, HbA1c, and TG, and negatively correlated with IL-10. L-Lysine was negatively correlated with FBG, FINS, and HbA1c. L- Tryptophan was positively correlated with IL-10, and negatively correlated with FBG, HbA1c, FINS, LDL, and TG. L-Valine was positively correlated with FBG, FINS, HbA1c, and LDL, and negatively correlated with IL-10. N-Acetylornithine was positively correlated with FBG, FINS, HbA1c, LDL, TG, and TC, and negatively correlated with IL-10. Pyruvic acid was positively correlated with IL-10, and negatively correlated with FINS, FBG, HbA1c, and LDL. Serotonin was positively correlated with FBG, FINS, and TG, and negatively correlated with IL-10.

Thirteen JTP-regulated potential biomarkers were correlated with the 13 differential gut microbiota using spearman calculations (Figure 6B). Positive correlations are shown in red, negative correlations in blue, and those with *p* < 0.05 and | r| > 0.5 were selected as correlating.

At the phylum level, the Actinobacteria were positively correlated with N-Acetylornithine, L-Methionine, L-Valine, 3-Hydroxybutyric acid, Choline, L-Aspartic acid, Serotonin, and negatively correlated with Arachidonic acid, L-Tryptophan, L-Lysine, Pyruvic acid, L-Histidine. Bacteroidetes were negatively correlated with L-Histidine, Pyruvic acid. Firmicutes were positively correlated with L-Histidine, Pyruvic acid, and Arachidonic acid and negatively correlated with L-Methionine. Proteobacteria were positively correlated with Serotonin, L-Valine, N-Acetylornithine, 3-Hydroxybutyric acid, L-Methionine, and Choline, and negatively correlated with L-Tryptophan, L-Histidine, Arachidonic acid, L-Lysine, Pyruvic acid was negatively correlated.

At the genus level, the Clostridium_IV was positively correlated with Arachidonic acid, L-Tryptophan, Pyruvic acid, and L-Histidine, and negatively correlated with L-Valine, N-Acetylornithine, 3-Hydroxybutyric acid, Serotonin, and L-Methionine were negatively correlated. Clostridium_XlVa was positively correlated with Pyruvic acid, L-Histidine, Arachidonic acid, and L-Tryptophan and negatively correlated with 3-Hydroxybutyric acid. Eubacterium was positively correlated with L-Lysine. Fusicatenibacter was positively correlated with L-Methionine, Serotonin, 3-Hydroxybutyric acid, and N-Acetylornithine, and negatively correlated with L-Histidine, L-Lysine, Pyruvic acid, L-Alanine, L-Tryptophan, and Arachidonic acid. Romboutsia was positively correlated with L-Valine and N-Acetylornithine and negatively correlated with L-Tryptophan, Arachidonic acid, Pyruvic acid, and L-Lysine. Roseburia was positively correlated with L-Histidine, Arachidonic acid, Pyruvic acid, and L-Tryptophan, and negatively correlated with 3-Hydroxybutyric acid, Serotonin, L-Methionine, N-Acetylornithine, L-Valine.

At the family level, the Eubacteriaceae were positively correlated with L-Lysine. Prevotellaceae was positively correlated with L-Methionine, and negatively correlated with L-Histidine, L-Lysine, Pyruvic acid, and Arachidonic acid. Ruminococcaceae was positively correlated with Arachidonic acid, L-Tryptophan, and negatively correlated with N-Acetylornithine, 3-Hydroxybutyric acid, Serotonin, L-Methionine, L-Valine.

The biomarkers and gut microbiota with correlation were correlated with pharmacological indicators, and the Sankey diagram (Figure 6C) shows that 7 pharmacological indicators, 12 biomarkers, and 11 gut microbiota were highly correlated.

## 4. Discussion

Type 2 diabetes mellitus model rats established by high-fat diet and low dose STZ had dry, gray, and loose hair, depression, gradual loss of body shape, irritability, high urine output, and sour and pungent taste. The study showed that the rats fed with high-fat diets exhibited insulin resistance, increased pancreatic islet β cells, and impaired islet β-cell function (15). It was consistent with the results of HE staining of pancreatic tissues. In the study, it was found that the levels of FBG, HbA1c, and FINS were increased in the model rats, which were consistent with the clinical manifestations of hyperglycemia and insulin resistance in type 2 diabetes mellitus. The levels of TC, TG, and LDL were increased in the model rats, which were consistent with the manifestation of dyslipidemia in type 2 diabetes mellitus. IL-10, which is involved in the regulation of insulin secretion, β-cell apoptosis, and peripheral insulin resistance, was reduced in the T2DM model. The successful establishment of the T2DM model was confirmed by a combination of multiple aspects. JTP reversed the symptoms of hyperglycemia, insulin resistance, dyslipidemia, and tissue damage. The efficacy of JTP in the treatment of type 2 diabetes mellitus was demonstrated.

Based on 16S rRNA sequencing analysis, we observed a decrease in alpha diversity of gut microbiota in T2DM group rats through sobs, Chao, Shannon, and ace indexes. Microbial species diversity is the basis of intestinal nutrient absorption, metabolism, and immune barrier regulation (16), and a decrease in flora species diversity affects intestinal function and induces various diseases. In the JTP group, the above indices increased, the diversity of flora increased, and the gut microbiota disorder was restored. Alterations in the gut microbiota were closely associated with T2DM, consistent with previous findings (17, 18). In this study, PCoA and NMDS analysis were used to analyze the similarity (β-diversity) of the gut microbiota of the samples. Two-dimensional plots of both PCoA and NMDS showed that samples from the T2DM group were far from the other three groups, and samples from the control and T2DM groups clustered on the left and right sides of the horizontal axis, respectively. The samples of the JTP group and MET group were distributed on the side of the control group, indicating that the structure of intestinal microflora after JTP and MET treatment was more similar to that of the control group.

In our study, the dominant microorganisms in the rat gut mainly included Firmicutes, Bacteroidetes, Proteobacteria, and Actinobacteria. The stability of these flora plays an important role in immune regulation, energy metabolism, and substance metabolism, which are essential factors for the maintenance of human health and a mirror reflecting the internal environment of the organism (19). Compared to the control group, the relative abundance of Actinobacteria, Proteobacteria, and Bacteroidetes in the gut microbiota was increased and the relative abundance of Firmicutes was decreased in the T2DM group. Carbohydrates and proteins in the gut are metabolized and hydrolyzed primarily by Firmicutes, whereas steroids, polysaccharides, and bile acids are metabolized by Bacteroidetes (20, 21). Studies have shown that short-chain fatty acids not only provide energy for intestinal epithelial cells but also enhance the intestinal defense barrier. Firmicutes are the main species that ferment carbohydrates into various short-chain fatty acids (21). As the abundance of Firmicutes decreases, these protective effects are weakened, which is likely to induce the occurrence of type 2 diabetes mellitus. The increase in abundance of Bacteroidetes and the disturbance of lipid and energy metabolism further accelerate gut microbiota disorders and contribute to the development of type 2 diabetes mellitus (22). Bacteroidetes accelerate the secretion of lipopolysaccharides and thus cause insulin resistance (23). The increased abundance of Actinobacteria and Proteobacteria in the T2DM group, with gut microbiota imbalance, triggers increased intestinal wall permeability which allows a large number of intestinal bacteria to translocate and distribute in blood and tissues, causing insulin resistance.

The study also confirmed that the abundance of Gram-negative pathogenic bacteria (family level: Prevotellaceae; genus level: Romboutsia, Desulfovibrio)was increased when type 2 diabetes mellitus occurred. Gram-positive bacteria (family level: Eubacteriaceae, Ruminococcaceae; genus level: Clostridium_IV, Clostridium_XlVa, Eubacterium, Roseburia) decreased in abundance. The increase of Gram-negative bacteria will cause an increase in cytosolic toxicogenic LPS (24). In particular, there is an increase in the abundance of endotoxin-producing Desulfovibrio (Desulfovibrio), which impairs intestinal barrier function and leads to high levels of circulating LPS (25). The reduction of beneficial bacteria such as Clostridium increases intestinal permeability. At this point, gut microbiota toxins enter the circulatory system, causing inflammation and then leading to insulin resistance.

PICRUST analysis revealed that gut microbiota is involved in metabolic pathways such as amino acid metabolism, carbohydrate metabolism, and lipid metabolism affecting type 2 diabetes mellitus. Metabolomics was used to further analyze the metabolism of rats. The JTP callback of metabolic pathways associated with type 2 diabetes mellitus is described below.

Metabolomics studies have shown that T2DM rats are always accompanied by abnormal amino acid metabolism. L-Phenylalanine and L-Tryptophan belong to the aromatic amino acids (AAA), which affect insulin resistance and dyslipidemia, and play an important role in type 2 diabetes mellitus (26). L-Phenylalanine is involved in glucose and fat metabolism in the body through oxidative conversion to tyrosine catalyzed by phenylalanine hydroxylase. It has been reported that L-Phenylalanine levels are elevated in type 2 diabetic patients and T2DM rat models (27). This is consistent with the results of our results that the level of L-Phenylalanine in feces of T2DM group increased. L-Tryptophan is an important metabolite involved in the regulation of inflammation and may reduce blood glucose levels in T2DM rats by relieving inflammation and promoting insulin sensitivity (28, 29). Gut microbiota can decompose tryptophan to produce indole, and long-term increased indole levels can inhibit mitochondrial metabolism, reduce intracellular ATP concentration, and reduce ATP-sensitive potassium channel opening. This inhibits the secretion of gastrointestinal hormones such as pancreatic hyperglycemic polypeptide (30). In the T2DM model group, Actinobacteria, Proteobacteria, Fusicatenibacter, and Romboutsia were elevated in abundance, and L-Tryptophan and Pyruvic acid levels decreased, which were correlated and negatively correlated. The levels of L-Phenylalanine and L-Tryptophan were increased after JTP treatment. Valine belongs to the branched chain amino acids (BCAA). In our study, it was higher in the T2DM model group than in the control group. It was shown that increased BCAA levels can lead to increased insulin secretion and islet β-cell depletion in T2DM patients (31). Abnormal BCAA metabolism can lead to the accumulation of valine, resulting in β-cell mitochondrial dysfunction and high sensitivity to insulin resistance. It can be used as a characteristic biomarker of type 2 diabetes mellitus. Aspartate and glutamate metabolism can inhibit Akt phosphorylation (32), and activation of Akt phosphorylated insulin promotes glycogen synthesis and inhibits gluconeogenesis in the liver, thereby lowering blood glucose (33). L-Aspartic acid and L-Glutamic acid, as core aspartate and glutamate metabolism metabolites, were increased in the T2DM model group. Histidine metabolism is a key metabolic pathway affecting type 2 diabetes mellitus, and L-Histidine is a core metabolite of histidine metabolism. Histidine supplementation has been found to suppress the inflammatory response and improve insulin resistance. L-Lysine significantly improves the structure and function of glycosylated lysozyme *in vitro* and is an effective therapeutic supplement for T2DM (34). L-Histidine and L-Lysine levels are decreased in the T2DM model. l-Methionine undergoes demethylation and further hydrolysis to form cysteine. Agullo-Ortuno et al found that homocysteine levels are an indicator of diabetes risk (35). In this study, L-Methionine levels were increased in the T2DM model group. In summary, the regulation of amino acid metabolism may have significant implications for the treatment of diabetes, and JTP plays a therapeutic role in type 2 diabetes mellitus by regulating Tryptophan metabolism, Alanine, aspartate and glutamate metabolism, Histidine metabolism, Cysteine and methionine metabolism.

Carbohydrate metabolism was significantly altered in T2DM rats. Pyruvic acid is the end product of glycolysis and the starting point for gluconeogenesis, and can be generated by transamination and participate in energy metabolism to provide energy to living cells and organisms (36, 37). The pyruvate dehydrogenase complex converts Pyruvic acid to acetyl coenzyme A and enters the tricarboxylic acid cycle (38).

At the same time, Pyruvic acid can be fermented into succinate, lactate, and acetyl-CoA by gut microbiota and further metabolized into SCFAs. This affects changes in levels of glycolysis/gluconeogenesis and pyruvate metabolism (39). In this study, Firmicutes was found to be positively correlated with Pyruvic acid, and Actinobacteria, Bacteroidetes, and Proteobacteria were negatively correlated with Pyruvic acid. Pyruvic acid levels are decreased and energy metabolism is disturbed in diabetic patients. Pyruvic acid levels reverted to normal after JTP treatment.

Lipid metabolism is highly associated with the development of type 2 diabetes mellitus (T2DM). Arachidonic acid is a polyunsaturated fatty acid that inhibits lipogenesis and promotes lipolysis (40)and plays an important role in regulating lipid metabolism in the body (41). A decrease in Arachidonic acid has been reported to affect lipid metabolism disorders in type 2 diabetes mellitus. At the same time, our experimental results showed that TC, TG, and LDL levels were increased in T2DM model rats, which is consistent with previous reports. Arachidonic acid is present in the structural phospholipids of cell membranes (42)and delays the progression of type 2 diabetes mellitus by increasing the fluidity of cell membranes and increasing the number of insulin receptors and their affinity for insulin. Our study found a positive correlation between Ruminococcaceae and Arachidonic acid, consistent with previous studies (43). Ruminococcaceae produces SCFAs that stimulate glucagon secretion and increase satiety, thereby regulating fat and cholesterol synthesis and regulating lipid metabolism in humans (44). After 4 weeks of JTP treatment, the level of Arachidonic acid in feces increased and the abundance of Ruminococcaceae increased, suggesting that JTP improves type 2 diabetes mellitus by regulating Arachidonic acid and Ruminococcaceae to improve lipid metabolism disorders, inflammation, and insulin secretion.

This study mainly focuses on the changes in gut microbiota and metabolite detection in JTP intervention in T2DM rats. The animal indicators are currently limited to the phenotypic part. In the future, experiments are needed to further improve the exploration of pathways and mechanisms.

## 5. Conclusion

In summary, Jiaotai pill (JTP) decreased blood glucose and lipid levels and reduced insulin resistance in type 2 diabetes mellitus (T2DM) rats. Potential biomarkers and key functional bacteria were identified for JTP treatment in T2DM rats. These results suggest that JTP can increase microbiota diversity and restore gut microbiota balance. It improves metabolic pathways such as amino acid metabolism, carbohydrate metabolism, and lipid metabolism associated with type 2 diabetes mellitus, and plays a therapeutic role in T2DM rats.

## Data availability statement

The datasets presented in this study can be found in online repositories. The name of the repository and accession number can be found below: https://www.ncbi.nlm.nih.gov/bioproject/922696.

## Ethics statement

This animal study was reviewed and approved. All experiments were performed following the Declaration of Helsinki and were approved by the Animal Health and Ethics Committee of Heilongjiang University of Chinese Medicine (2021012709).

## Author contributions

XhW and JL designed the study. XW conducted the animal trial, sample collection, and physical analysis. QL and SY performed the bioinformatics analysis of 16S rRNA sequencing. JL and YZ performed the untargeted metabolomics data. JL and CP wrote the manuscript. XhW, JL, and ZF contributed to the discussion of the work and assisted in drafting the manuscript. All authors read and approved the final manuscript.

## Footnotes

1
https://www.metaboanalyst.ca/
