# Supplementary material for: Fecal metabolomics combined with 16S rRNA gene sequencing to analyze the effect of Jiaotai pill intervention in type 2 diabetes mellitus rats
Source: Front Nutr. 2023 May 9;10:1135343. doi: 10.3389/fnut.2023.1135343 (PMC10203393; doi:10.3389/fnut.2023.1135343)
Supplement: Supplementary file 1 [file Data_Sheet_1.docx]

Supplementary Material

Fecal metabolomics combined with 16S rRNA gene sequencing to analyze the effect of Jiao-Tai pill intervention in type 2 diabetes mellitus rats

**Jing Liu^1^, Xu Wang^2^, Qiyao Li^1^, Chengyu Piao^2^, Zuowang Fan^1^, Yao Zhang^1^, Saisai Yang^1^, Xiuhong Wu^1*^**

*** Correspondence: Xiuhong Wu**: wxh8088@163.com

# Supplementary Figures and Tables

## Supplementary Figures


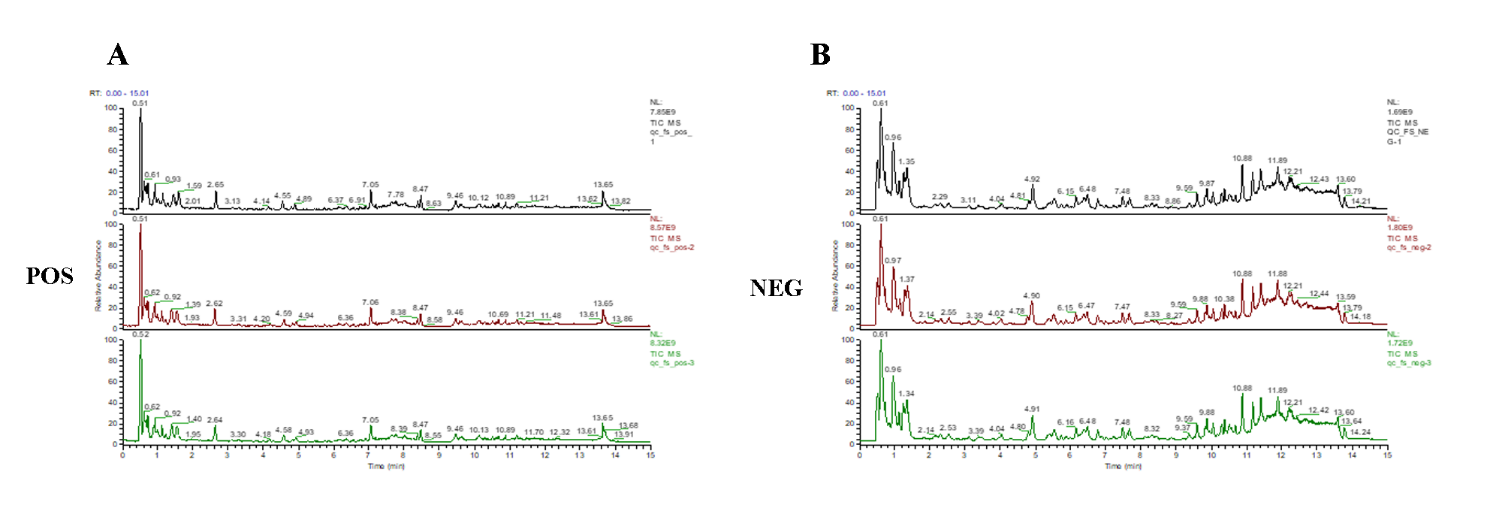


**Supplementary Figure 1.** (A). Total Ion Chromatography (TIC) of quality control (QC) samples were obtained in positive ion mode. (B). Total Ion Chromatography (TIC) of quality control (QC) samples were obtained negative ion mode.


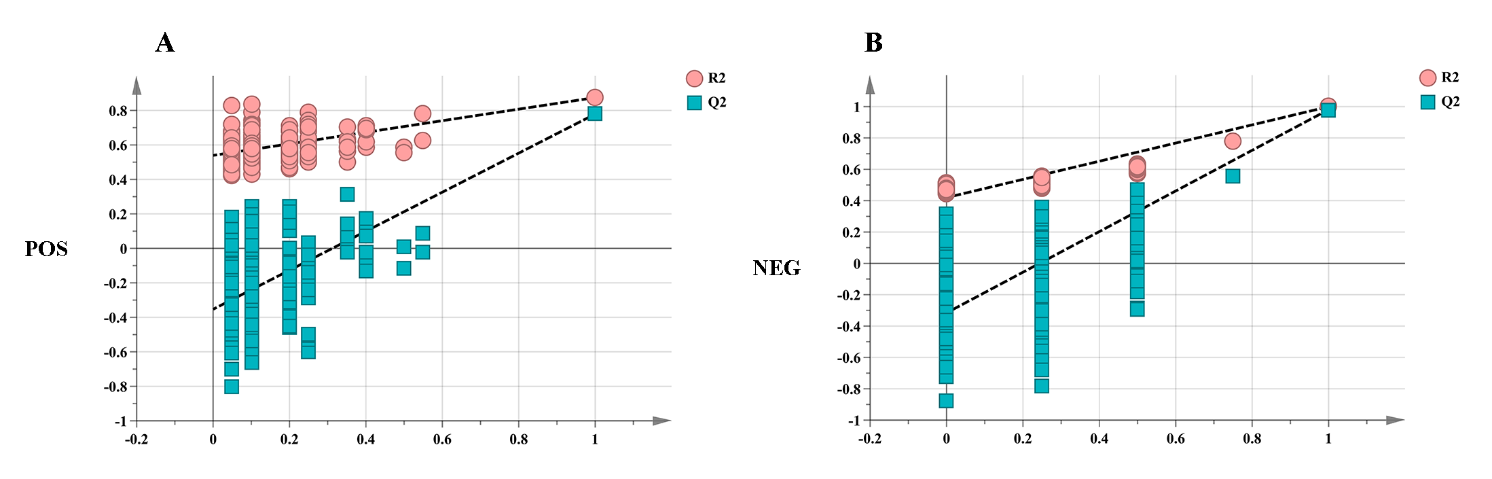


**Supplementary Figure 2.** (A). Statistical validation of the OPLS-DA model using permutation analysis in positive ion mode. (B). Statistical validation of the OPLS-DA model using permutation analysis in positive ion mode in negative ion mode.


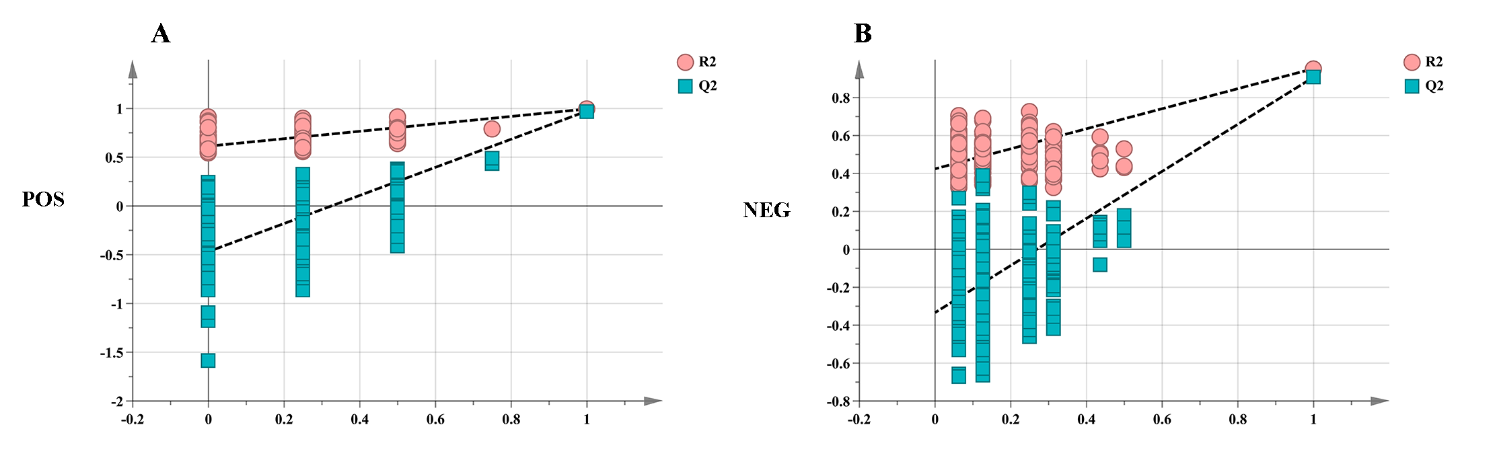


**Supplementary Figure 3.** (A). Statistical validation of the PLS-DA model using permutation analysis in positive ion mode. (B) Statistical validation of the PLS-DA model using permutation analysis in positive ion mode in negative ion mode.

## Supplementary Tables

Supplementary Table1. Specific information of potential biomarkers relevant to T2DM model rats.

| **No.** | **Rt/min** | **Mass** | **Calc Mass** | **Adducts** | **ppm** | **Proposed Composition** | **Postulated Identity** | **HMDB** | **Trend** | **JTP** |
| --- | --- | --- | --- | --- | --- | --- | --- | --- | --- | --- |
| 1 | 2.640 | 165.07898 | 165.07899 | M+H | 0.08 | C_9_H_11_NO_2_ | L-Phenylalanine | HMDB0000159 | ↓^**^ | + |
| 2 | 0.738 | 117.07898 | 117.07898 | M+H | 0.02 | C_5_H_11_NO_2_ | L-Valine | HMDB0000883 | ↑^**^ | +^##^ |
| 3 | 0.616 | 103.09971 | 103.09966 | M+H | -0.51 | C_5_H_13_NO | Choline | HMDB0000097 | ↑^**^ | +^##^ |
| 4 | 0.607 | 133.03751 | 133.03753 | M+H | 0.14 | C_4_H_7_NO_4_ | L-Aspartic acid | HMDB0000191 | ↑^*^ | +^#^ |
| 5 | 2.502 | 176.09496 | 176.09504 | M+H | 0.44 | C_10_H_12_N_2_O | Serotonin | HMDB0000259 | ↑^**^ | +^#^ |
| 6 | 1.441 | 141.09021 | 141.09033 | M+H | 0.83 | C_6_H_11_N_3_O | L-Histidinol | HMDB0003431 | ↓^**^ | + |
| 7 | 0.941 | 132.05349 | 132.05359 | M+H | 0.74 | C_4_H_8_N_2_O_3_ | Ureidopropionic acid | HMDB0000026 | ↑^*^ | + |
| 8 | 0.871 | 105.04259 | 105.04260 | M+H | 0.11 | C_3_H_7_NO_3_ | D-Serine | HMDB0003406 | ↓^**^ | + |
| 9 | 4.008 | 204.08988 | 204.08980 | M+H | -0.4 | C_11_H_12_N_2_O_2_ | D-Tryptophan | HMDB0013609 | ↓^**^ | + |
| 10 | 0.611 | 146.06914 | 146.06915 | M+H | 0.02 | C_5_H_10_N_2_O_3_ | L-Glutamine | HMDB0000641 | ↑^**^ | - |
| 11 | 0.557 | 146.10553 | 146.10558 | M+H | 0.38 | C_6_H_14_N_2_O_2_ | L-Lysine | HMDB0000182 | ↓^**^ | +^#^ |
| 12 | 7.628 | 155.06948 | 155.06961 | M-H | 0.86 | C_6_H_9_N_3_O_2_ | L-Histidine | HMDB0000177 | ↓^**^ | +^##^ |
| 13 | 0.615 | 119.05824 | 119.05830 | M-H | 0.50 | C_4_H_9_NO_3_ | L-Threonine | HMDB0000167 | ↓^**^ | + |
| 14 | 0.632 | 147.05316 | 147.05327 | M-H | 0.78 | C_5_H_9_NO_4_ | L-Glutamic acid | HMDB0000148 | ↑^**^ | - |
| 15 | 5.109 | 104.04734 | 104.04734 | M-H | -0.05 | C_4_H_8_O_3_ | 3-Hydroxybutyric acid | HMDB0000357 | ↑^**^ | +^##^ |
| 16 | 0.810 | 88.01604 | 88.01608 | M-H | 0.37 | C_3_H_4_O_3_ | Pyruvic acid | HMDB0000243 | ↓^**^ | +^##^ |
| 17 | 0.718 | 192.02700 | 192.02723 | M-H | 1.18 | C_6_H_8_O_7_ | Citric acid | HMDB0000094 | ↑^**^ | + |
| 18 | 0.768 | 174.10044 | 174.10056 | M-H | 0.69 | C_7_H_14_N_2_O_3_ | N-Acetylornithine | HMDB0003357 | ↑^**^ | +^##^ |
| 19 | 1.216 | 116.01096 | 116.01095 | M-H | -0.05 | C_4_H_4_O_4_ | Fumaric acid | HMDB0000134 | ↑^**^ | + |
| 20 | 11.826 | 304.24023 | 304.24048 | M-H | 0.82 | C_20_H_32_O_2_ | Arachidonic acid | HMDB0001043 | ↓^**^ | +^#^ |
| 21 | 3.856 | 204.08988 | 204.09016 | M-H | 1.38 | C_11_H_12_N_2_O_2_ | L-Tryptophan | HMDB0000929 | ↓^**^ | +^#^ |
| 22 | 0.913 | 89.04768 | 89.04770 | M-H | 0.26 | C_3_H_7_NO_2_ | L-Alanine | HMDB0000161 | ↓^*^ | +^#^ |
| 23 | 0.995 | 149.05105 | 149.05119 | M-H | 0.94 | C_5_H_11_NO_2_S | L-Methionine | HMDB0000696 | ↑^**^ | +^##^ |

Note: ↑↓indicates the level of biomarkers in T2DM model group increased or decreased;+& - indicates whether the drug has a callback effect on the marker; Compared with the blank control group, **p*<0.05, ***p*<0.01;Compared with the model group, ^#^*p*<0.05, ^##^*p*<0.01.

Supplementary Table2. Secondary fragmentation information of potential biomarkers relevant to T2DM model rats.

| **No** | **Rt/min** | **Mass** | **Adducts** | **Proposed**  **Composition** | **Postulated Identity** | **MS/MS fragmention(m/z)** |
| --- | --- | --- | --- | --- | --- | --- |
| 1 | 2.640 | 165.07898 | M+H | C_9_H_11_NO_2_ | L-Phenylalanine | 166.08626[M+H]^+^, 149.05972[M+H-NH_3_]^+^, 131.04914[M+H-NH_5_O]^+^, 120.08081[M+H-CH_2_O_2_]^+^, 107.04914[M+H-C_2_H_5_NO]^+^, 103.05423[M+H-CH_5_NO_2_]^+^, 93.06988[M+H-C_2_H_3_NO_2_]^+^, 91.05422[M+H-C_2_H_5_NO_2_]^+^, 79.05422[M+H-C_3_H_5_NO_2_]^+^, 77.03839[M+H-C_3_H_7_NO_2_]^+^, 53.03894[M+H-C_5_H_7_NO_2_]^+^ |
| 2 | 0.738 | 117.07898 | M+H | C_5_H_11_NO_2_ | L-Valine | 118.08626[M+H]^+^, 72.08078[M+H-CH_2_O_2_]^+^, 55.05423[M+H-CH_5_NO_2_]^+^ |
| 3 | 0.616 | 103.09971 | M+H | C_5_H_13_NO | Choline | 104.10699[M+H]^+^, 60.08078[M+H-C_2_H_4_O]^+^, 58.06513[M+H-C_2_H_6_O]^+^ |
| 4 | 0.607 | 133.03751 | M+H | C_4_H_7_NO_4_ | L-Aspartic acid | 134.04479[M+H]^+^, 156.02679[M+Na]^+^, 116.03422[M+H-H_2_O]^+^, 88.03930[M+H-CH_2_O_2_]^+^, 74.02365[M+H-C_2_H_4_O_2_]^+^, 70.02874[M+H-CH_4_O_3_]^+^ |
| 5 | 2.502 | 176.09496 | M+H | C_10_H_12_N_2_O | Serotonin | 177.10195[M+H]^+^, 160.07569[M+H-NH_3_]^+^, 142.06512[M+H-NH_5_O]^+^, 132.08078[M+H-CH_3_NO]^+^ |
| 6 | 1.441 | 141.09021 | M+H | C_6_H_11_N_3_O | L-Histidinol | 142.09749[M+H]^+^, 125.02353[M+H-NH_3_]^+^, 83.04923[M+H-C_2_H_5_NO]^+^, 60.25756[M+H-C_4_H_6_N_2_]^+^, 69.06998[M+H-C_3_H_7_NO]^+^ |
| 7 | 0.941 | 132.05349 | M+H | C_4_H_8_N_2_O_3_ | Ureidopropionic acid | 133.06087[M+H]^+^, 115.05020[M+H-C_2_H_2_O]^+^, 90.05495[M+H-CHNO]^+^ |
| 8 | 0.871 | 105.04259 | M+H | C_3_H_7_NO_3_ | D-Serine | 106.04987[M+H]^+^, 88.03930[M+H-H_2_O]^+^, 70.02874[M+H-H_4_O_2_]^+^, 60.04439[M+H-CH_2_O_2_]^+^ |
| 9 | 4.008 | 204.08988 | M+H | C_11_H_12_N_2_O_2_ | D-Tryptophan | 205.09702[M+H]^+^, 188.07097[M+H-NH_3_]^+^, 146.96169[M+H-C_2_H_5_NO]^+^, 118.06516[M+H-C_3_H_5_NO_2_]^+^ |
| 10 | 0.611 | 146.06914 | M+H | C_5_H_10_N_2_O_3_ | L-Glutamine | 147.07643[M+H]^+^, 130.04987[M+H-NH_3_]^+^, 101.07094[M+H-CH_2_O_2_]^+^, 102.05495[M+H-CH_3_NO]^+^, 84.04439[M+H-CH_5_NO_2_]^+^, 56.04948[M+H-C_2_H_5_NO_3_]^+^ |
| 11 | 0.557 | 146.10553 | M+H | C_6_H_14_N_2_O_2_ | L-Lysine | 147.11280[M+H]^+^, 130.08626[M+H-NH_3_]^+^, 129.10226[M+H-H_2_O]^+^, 56.04948[M+H-C_3_H_9_NO_2_]^+^, 67.05423[M+H-CH_8_N_2_O_2_]^+^, 84.08078[M+H-CH_5_NO_2_]^+^ |
| 12 | 7.628 | 155.06948 | M-H | C_6_H_9_N_3_O_2_ | L-Histidine | 154.06220[M-H]^-^, 137.03565[M-H-NH_3_]^-^, 110.07237[M-H-CO_2_]^-^, 108.05672[M-H-CH_2_O_2_] ^-^, 93.04582[M-H-CNH_3_O_2_] ^-^, 72.00910[M-H-C_4_H_6_N_2_]^-^, 81.04582[M-H-C_2_H_3_NO_2_] ^-^，67.03017[M-H-C_2_H_3_N_2_O_2_] ^-^ |
| 13 | 0.615 | 119.05824 | M-H | C_4_H_9_NO_3_ | L-Threonine |  |
| 14 | 0.632 | 147.05316 | M-H | C_5_H_9_NO_4_ | L-Glutamic acid | 146.04588[M-H]^-^, 128.03532[M-H-H_2_O]^-^, 102.05605[M-H]^-^ |
| 15 | 5.109 | 104.04734 | M-H | C_4_H_8_O_3_ | 3-Hydroxybutyric acid | 103.04007[M-H]^-^, 59.01385[M-H-CO_2_]^-^ |
| 16 | 0.810 | 88.01604 | M-H | C_3_H_4_O_3_ | Pyruvic acid | 87.00877[M-H]^-^, 59.01411[M-H-CO]^-^ |
| 17 | 0.718 | 192.02700 | M-H | C_6_H_8_O_7_ | Citric acid | 191.01971[M-H]^-^, 173.00893[M-H-H_2_O]^-^, 129.01924[M-H-CH_2_O_3_]^-^, 111.00874[M-H-CH_4_O_4_]^-^, 87.00874[M-H-C_3_H_4_O_4_]^-^, 85.02950[M-H-C_2_H_2_O_5_]^-^ |
| 18 | 0.768 | 174.10044 | M-H | C_7_H_14_N_2_O_3_ | N-Acetylornithine | 173.09317[M-H]^-^, 131.08260[M-H-C_2_H_2_O]^-^ |
| 19 | 1.216 | 116.01096 | M-H | C_4_H_4_O_4_ | Fumaric acid | 115.00368[M-H]^-^, 71.01385[M-H-CO_2_]^-^ |
| 20 | 11.826 | 304.24023 | M-H | C_20_H_32_O_2_ | Arachidonic acid | 303.23295[M-H]^-^, 259.24312[M-H-C_2_H_4_O]^-^, 205.19618[M-H-C_5_H_6_O_2_]^-^, 59.01365[M-H-C_17_H_240_]^-^ |
| 21 | 3.856 | 204.08988 | M-H | C_11_H_12_N_2_O_2_ | L-Tryptophan | 203.08260[M-H]^-^, 142.06622[M-H-CH_3_NO_2_]^-^, 116.05057[M-H-C_3_H_5_NO_2_]^-^, 74.02475[M-H-C_9_H_7_N]^-^, 72.00910[M-H-]^-^ |
| 22 | 0.913 | 89.04768 | M-H | C_3_H_7_NO_2_ | L-Alanine | 88.04040[M-H]^-^, 71.01385[M-H-NH_3_]^-^, 60.00910[M-H-C_2_H_4_]^-^, 59.01385[M-H-CH_3_N]^-^ |
| 23 | 0.995 | 149.05105 | M-H | C_5_H_11_NO_2_S | L-Methionine | 148.04407[M-H]^-^, 105.03464[M-H-CHON]^-^, 100.04002[M-H-SO]^-^, 59.75528[M-H-C_4_H_10_ON]^-^ |

Supplementary Table3. Specific information of Metabolism pathway with 23 potential biomarkers relevant to T2DM rats.

| **Pathway** | **Total** | **Expected** | **Hits** | **Raw p** | **FDR** | **Impact** |
| --- | --- | --- | --- | --- | --- | --- |
| Alanine, aspartate and glutamate metabolism | 28 | 0.389660 | 7 | 3.32E-08 | 1.4E-06 | 0.53686 |
| D-Glutamine and D-glutamate metabolism | 6 | 0.083499 | 2 | 0.00268 | 0.02044 | 0.50000 |
| Phenylalanine, tyrosine and tryptophan biosynthesis | 4 | 0.055666 | 1 | 0.05457 | 0.30558 | 0.50000 |
| Phenylalanine metabolism | 12 | 0.167000 | 1 | 0.15531 | 0.56724 | 0.35714 |
| Arachidonic acid metabolism | 36 | 0.500990 | 1 | 0.39980 | 1.00000 | 0.33292 |
| Tryptophan metabolism | 41 | 0.570580 | 2 | 0.10931 | 0.45535 | 0.24798 |
| Histidine metabolism | 16 | 0.222660 | 3 | 0.00116 | 0.01623 | 0.22131 |
| Pyruvate metabolism | 22 | 0.306160 | 2 | 0.03605 | 0.21628 | 0.20684 |
| Citrate cycle (TCA cycle) | 20 | 0.278330 | 3 | 0.00228 | 0.02044 | 0.16653 |
| Arginine biosynthesis | 14 | 0.194830 | 5 | 5.8E-07 | 1.63E-05 | 0.11675 |
| beta-Alanine metabolism | 21 | 0.292250 | 3 | 0.00263 | 0.02044 | 0.10448 |
| Cysteine and methionine metabolism | 33 | 0.459240 | 2 | 0.07520 | 0.37155 | 0.10446 |
| Glycolysis / Gluconeogenesis | 26 | 0.361830 | 1 | 0.30750 | 0.95666 | 0.10044 |
| Arginine and proline metabolism | 38 | 0.528830 | 2 | 0.09606 | 0.44398 | 0.08600 |
| Glyoxylate and dicarboxylate metabolism | 32 | 0.445330 | 4 | 0.00078 | 0.01623 | 0.03175 |
| Pantothenate and CoA biosynthesis | 19 | 0.264410 | 3 | 0.00195 | 0.02044 | 0.02857 |
| Glycerophospholipid metabolism | 36 | 0.500990 | 1 | 0.39980 | 1.00000 | 0.02582 |
| Tyrosine metabolism | 42 | 0.584490 | 2 | 0.11384 | 0.45535 | 0.02463 |
| Glycine, serine and threonine metabolism | 34 | 0.473160 | 4 | 0.00098 | 0.01623 | 0.02408 |
| Glutathione metabolism | 28 | 0.389660 | 1 | 0.32698 | 0.98094 | 0.01966 |
| Pyrimidine metabolism | 39 | 0.542740 | 2 | 0.10042 | 0.44398 | 0.01318 |
| Aminoacyl-tRNA biosynthesis | 48 | 0.667990 | 11 | 2.84E-12 | 2.39E-10 | 0 |
| Nitrogen metabolism | 6 | 0.083499 | 2 | 0.00267 | 0.02044 | 0 |
| Valine, leucine and isoleucine biosynthesis | 8 | 0.111330 | 2 | 0.00491 | 0.03439 | 0 |
| Butanoate metabolism | 15 | 0.208750 | 2 | 0.01737 | 0.11225 | 0 |
| Synthesis and degradation of ketone bodies | 5 | 0.069583 | 1 | 0.06776 | 0.35574 | 0 |
| Biotin metabolism | 10 | 0.139170 | 1 | 0.13113 | 0.50069 | 0 |
| Nicotinate and nicotinamide metabolism | 15 | 0.208750 | 1 | 0.19039 | 0.66636 | 0 |
| Selenocompound metabolism | 20 | 0.278330 | 1 | 0.24578 | 0.82584 | 0 |
| Lysine degradation | 25 | 0.347910 | 1 | 0.29756 | 0.95666 | 0 |
| Porphyrin and chlorophyll metabolism | 30 | 0.417500 | 1 | 0.34594 | 1.00000 | 0 |
| Biosynthesis of unsaturated fatty acids | 36 | 0.500990 | 1 | 0.39980 | 1.00000 | 0 |
| Valine, leucine and isoleucine degradation | 40 | 0.556660 | 1 | 0.43334 | 1.00000 | 0 |
| Purine metabolism | 66 | 0.918490 | 1 | 0.61156 | 1.00000 | 0 |
